# With Appreciation

**DOI:** 10.1002/jcsm.12263

**Published:** 2017-11-23

We thank the following individuals who served as manuscript reviewers for The Journal of Cachexia, Sarcopenia and Muscle (JCSM) in 2017.

We highly appreciate their efforts. Fair, conscientious and timely peer reviews contribute to the success of JCSM. The Editors.

## 6 and more reviews

Vickie Baracos
Paola Costelli
Marcelo Dos Santos
Michiyoshi Hatanaka
Alexander Krannich
Alessandro Laviano

## 3–5 reviews

Eirik Aahlin
Volker Adams
Stephen E. Alway
Asfar Azmi
Thiago Barbosa‐Silva
Libera Berghella
James Carson
Sami Dridi
Nicole Ebner
Wei He
Jörg Heineke
Neil Johns
Mitja Lainscak
Lisa Martin
Emanuele Marzetti
Vera Mazurak
Evasio Pasini
Rosanna Piccirillo
Russ Price
Kunihiro Sakuma
Joerg Schefold
Tora Solheim
Yefei Wen
Antonio Zorzano

## 1–2 reviews

Santanasto Adam
Miguel Alonso‐Alonso
Haruka Amitani
Bradley Anawalt
Hidenori Arai
Victor Arce
Koji Ataka
Gulistan Bahat
Christine Baldwin
Ronald Barr
Esther Barreiro
Ivan Bautmans
Daniel Bechet
Srinivasan Beddhu
Martha Belury
David Berger
Cemil Bilir
Anne Blaes
Andrea Bonetto
Scott Bowen
Justin Brown
Gillian Butler‐Browne
Bette Caan
Patricia Campos‐Ferraz
Eli Carmeli
Carmen Castaneda Sceppa
David Cella
Shu‐Fang Chang
Martin Chasen
Stephanie Chevalier
John Cleland
Paul Coen
Dario Coletti
Lilian Cuppari
Frederic De Ceuninck
Maxine De La Cruz
Michael De Lisio
Marian de van der Schueren
Hans Degens
Egidio Del Fabbro
Patrice Delafontaine
Louise Deldicque
Rony Dev
Francesco Dioguardi
Wolfram Doehner
Cathleen Drescher
Carrie Earthman
Stefan Engeli
Rita Ferreira
Alberto Frisoli
Bruno Gagnon
Jose Garcia
Jack Garnham
Alexander Gasch
Cecilia Gelfi
Stephan Gielen
Ioannis Gioulbasanis
Francois Goldwasser
Maria Cristina Gonzalez
Harry Gosker
Paul Greenhaff
Bruno Gualano
Cheng Guo
Denis Guttridge
Dirk Habedank
Dietrich Hasper
David N Herndon
Stephan Herzig
David Hui
Sabah Hussain
Satoshi Ikeda
Marco Invernizzi
Aminah Jatoi
Nico Jehmlich
Achim Joerres
Kirsten Johansen
Toshimi Kaido
Carrie Karvonen‐Gutierrez
M.H.G. Katz
Kate Kennedy
Robert Kilgour
Yong‐Lim Kim
Serkan Kir
Brandon M. Kistler
Yann Klimentidis
Ger Koek
Hirotaka Konishi
Jeroen Kooman
Catherine Kubrak
Masafumi Kuzuya
Anis Larbi
Michaël Laurent
Susan Legrand
Andrew Lemmey
Paul Lewandowski
Jessica Lieffers
Kuan‐Yin Lin
Cheng‐Chieh Lin
Hui Ling
Jianming Liu
Maria Lorenzi
Antonio Macciò
Matthew Maddocks
Keisuke Maeda
Robert Mak
Theodore Malmstrom
Marwan Mannaa
Giovanni Mantovani
Daniel Marks
Hugo Maxwell Pereira
Ram Miller
William E. Mitch
Alessio Molfino
Aldo Montano‐Loza
Beatrice Morio
John Morley
Marina Mourtzakis
Andrew J. Murray
Maurizio Muscaritoli
Kirsten Ness
Anne Newman
Shu Nishiguchi
Enzo Nisoli
Toshimitsu Niwa
Kay Ohlendieck
Steven Olde Damink
Orestis Panagiotou
Jongha Park
Fabio Penna
Andrea Pereira
Goncalo Pereira
Stany Perkisas
Miroslav Petr
Martin Pichler
Patsie Polly
Carla Prado
Malte Puchert
Sarah Purcell
Zudin Puthuchaery
Joe Quadrilatero
Bruno Raynard
Sander Rensen
Connie Rhee
Linda Rinaman
Eric Roeland
Fernando Romeiro
Olav Rooyackers
Giuseppe Rosano
Ronenn Roubenoff
Karolina Rygiel
Michael Sawyer
Ralf Schindler
Gary Schwartz
Michael Schwarzer
Marília Seelaender
Cornel Sieber
Mario Siervo
Luis Simental‐Mendia
Andreas Simm
Alan Sinclair
Marianne Sinn
Jochen Springer
Marta Stelmach‐Mardas
Andrew Stewart Coats
Stephanie Studenski
Jukka Takala
Tetsuro Tamaki
Der‐Cherng Tarng
Juul J.W. Tegels
Jean‐Paul Thissen
Michael Toth
Klaske van Norren
Raymond Vanholder
Sudhakar Veeranki
Lex Verdijk
José M. Villalba
Dan Waitzberg
Hidetaka Wakabayashi
Stéphane Walrand
Jialin Wang
Adam Whaley‐Connell
Kenneth Wilund
Robert Wolfe
Rob Wust
Sumio Yamada
Carmine Zoccali

